# Monitoring Escape and Feeding Behaviours of Cruiser Fish by Inertial and Magnetic Sensors

**DOI:** 10.1371/journal.pone.0079392

**Published:** 2013-11-13

**Authors:** Takuji Noda, Yuuki Kawabata, Nobuaki Arai, Hiromichi Mitamura, Shun Watanabe

**Affiliations:** 1 Graduate School of Informatics, Kyoto University, Kyoto, Japan; 2 Institute for East China Sea Research, Nagasaki University, Nagasaki, Japan; 3 Field Science Education and Research Center, Kyoto University, Kyoto, Japan; University of California Davis, United States of America

## Abstract

A method was developed and applied for monitoring two types of fast-start locomotion (feeding and escape) of a cruiser fish, Japanese amberjacks *Seriola quinqueradiata*. A data logger, which incorporated a 3-axis gyroscope, a 3-axis accelerometer and a 3-axis magnetometer, was attached to the five fish. The escape, feeding and routine movements of the fish, which were triggered in tank experiments, were then recorded by the data logger and video cameras. The locomotor variables, calculated based on the high resolution measurements by the data logger (500 Hz), were investigated to accurately detect and classify the types of fast-track behaviour. The results show that fast-start locomotion can be detected with a high precision (0.97) and recall rate (0.96) from the routine movements. Two types of fast-start movements were classified with high accuracy (0.84). Accuracy was greater if the data were obtained from the data logger, which combined an accelerometer, a gyroscope and a magnetometer, than if only an accelerometer (0.80) or a gyroscope (0.66) was used.

## Introduction

Monitoring the locomotion and behaviour of animals is important for understanding their energy budgets and life histories. Especially, feeding and escape are two of the key behaviours for animals in terms of their survival. In aquatic animals, it has been demonstrated that many fish use fast-start (FS) locomotion, which involves a brief, sudden acceleration and turning for their escape and/or feeding; consequently, this type of locomotion has received significant attention in laboratory-based studies that use high-speed cameras [1]–[4]. However, the field measurements of feeding and escape behaviours have been usually challenging. Monitoring such behaviours in the field will reveal important insights into the physiological adaptation of animals to different environments and their survival strategies in a complex natural habitat, as well as the identification of ecologically important habitat (e.g. feeding ground).

The bio-logging method, which utilises data loggers attached to animals, has proven effective for the measurement of locomotion and behaviour of animals in the wild [5], [6]. In particular, data loggers with a 3-axis accelerometer and, in some cases, a 3-axis magnetometer have been used to monitor the attitude (pitch, roll and yaw) and dynamic acceleration of aquatic animals [7]–[9]. This type of information can be used to classify animal behaviour into categories such as swimming, resting, gliding, spawning, mating [9]–[13] and feeding [14], [15]. Recently, Bröell et al. [16] showed that different types of fast-start behaviours (feeding and escape) of a sit-and-wait predator *Myoxocephalus polyacanthocephalus* can be detected and identified with high accuracy (80%), using multiple locomotor variables obtained using accelerometers, given that its sampling frequency is sufficiently high (e.g. 100 Hz) to capture such agile movements [17]. Thus, data loggers can be useful for monitoring the fast-track behaviour of animals in the wild once the appropriate connection between the behaviour and the data logger measurements is established.

Fast-track behaviour of animals typically involves rapid turning and agile rotational movements [2]–[4]. Although many studies have used accelerometers to monitor the activity of animals in the wild [12], [13], [15], [16], this instrument alone is not sufficient to document the detailed 3D movements of animals because it is difficult to accurately differentiate gravity-based acceleration (which can be converted to pitch and roll) from dynamic acceleration [18], [19]. In addition, the accelerometer measurements do not provide rotational information, such as angular velocity and the direction of movement. Angular velocity can be directly measured with high temporal resolution (e.g. 100 Hz to 1 kHz) by a gyroscope. Therefore, using the gyroscope, if the initial attitude is known, any new attitude (hence, the gravity-based acceleration that would be measured by the accelerometer) can be estimated using initial attitude and the estimated attitude change calculated from the gyroscope measurements. An accelerometer and a magnetometer can be used in addition to a gyroscope to determine initial attitude and to correct the error when estimating attitude associated with the accumulation of noise from a gyroscope. In fact, it was shown that a novel gyroscope data logger (hereafter, gyro logger), which incorporates a 3-axis gyroscope, a 3-axis accelerometer and a 3-axis magnetometer (for a total of 9 axes), can reconstruct the fine-scale dynamic acceleration, gravity-based acceleration and attitude of animals (e.g. sea turtles), which have not been accurately measured in previous studies that only used an accelerometer (and occasionally a magnetometer) [19]. Therefore, a gyro logger may be more suitable for monitoring the fast-track behaviour of animals because this instrument can measure fine-scale dynamic acceleration and the angular velocity.

FS behaviour of fish has been intensively investigated in laboratory-based studies. In fish, there are various types of species which employ different locomotor modes (e.g. cruiser, saltatory, and sit-and-wait) and body forms (e.g. tunniform, carangiform, and anguilliform) adapted for their food search, predation and escape success in a complex natural habitat [20], [21], Obrien1990AS. These suggest that important locomotor variables for monitoring feeding and escape behaviours are different depending on the types of fish. While there is much research that investigated the locomotion of fish during their feeding and escape (e.g.[1]–[4]), little research has been conducted for cruiser fish [22], [23], that move continuously through their environment, searching constantly for their prey [21]. We have focused on cruiser fish, for which it may be difficult to identify the types of FS behaviour. It is unclear what kind of locomotor variables characterize the feeding and escape behaviours of cruiser fish, and which variables can be used for the identification of the behaviours using the measurements of their movements.

Given these considerations, the present study utilised a novel gyro logger for one of the cruiser fish, Japanese amberjacks *Seriola quinqueradiata* and investigated the following three questions; 1) Is it possible to use data logger measurements to accurately identify escape, feeding and routine movements (RM) of the fish? 2), If so, what types of locomotor variables obtained from the gyro logger measurements characterize the escape and feeding behaviours of the fish? 3) Is the accuracy better with the combined information from the gyroscope, accelerometer and magnetometer than with the information obtained using only an accelerometer or a gyroscope?

## Materials and Methods

### Ethics Statement

The care and sampling protocol for the tagging surgery and live predator-prey experiments in this study were approved by the Animal Research Committee of Kyoto University (permit number: Informatics 25-7).

### Study Animals

Five Japanese amberjacks [N = 5, fork length (FL): 65.9 ± 0.45 cm, body mass (BM): 4.08 ± 0.78 kg] (Table 1) were studied in tanks at the Institute for East China Sea Research at Nagasaki University in Japan. The feeding and escape movements of these animals were monitored by gyro loggers. The fish were obtained from a local fish hatchery (Nagasaki, Japan). The fish were maintained in a 300 cm diameter outdoor tank with flow-through seawater at a temperature of 19.05 ± 0.84°C, a depth of 1 m and a dissolved oxygen level of 84.89 ± 3.88%. The fish were acclimatised to the tank for at least one week prior to the initiation of the experiments, at which point each fish was tagged. After the fish were tagged, food was withheld to ensure a feeding response to the presence of live, wild-caught flathead silverside, *Hypoatherina valenciennei* [10 randomly selected samples; total length (TL) = 12.28 ± 0.60 cm], which were found to be one of the preferred prey of amberjacks through preliminary food selection trials using multiple types of natural prey.

**Table 1:** Summary of fish specifications and observed FS events.

fish ID	TL (cm)	FL (cm)	BM (kg)	N*_e_*	N*_f_*
**A**	71.6	66.8	3.2	11	10
**B**	66.6	60.9	4.1	8	11
**C**	69.8	63.9	3.8	11	20
**D**	77.8	73.1	5.3	4	0
**E**	67.8	65.0	3.9	7	0
			**total**	41	41

The total length (TL) and fork length (FL), the body mass (BM), and the number of escape (N***_e_***) and feeding (N***_f_***) movements that were identified using the video recordings of five (A–E) Japanese amberjacks.

### Data Logger

A gyro logger (LP-BLKU01, Biologging Solutions Inc., Kyoto, Japan), incorporating 3-axis accelerometer, 3-axis magnetometer and 3-axis gyroscope, was developed and used in this study. This data logger was cylindrical in shape (diameter: 3 cm, length: 17 cm) with a mass of 108 g in air, which included the attached CR123A battery. The measurement ranges were ±16 *g*, ±1.0 Gauss and ±1500 degree second^−1^, for the acceleration, magnetic field and angular velocity, respectively. The resolution of the measurements was 16 bit (−32768∼+32768). The data logger measured and stored all of the sensor outputs in an internal micro-SDHC memory (∼32 GB) at a sampling frequency of 500 Hz for a total sample time of 10 hours. Furthermore, this device allowed for multiple-scheduled recordings (e.g., 2 hours of recording each day). The data logger was covered with an alumite-treated case, which made it waterproof and pressure-proof up to a depth of 300 m. The logger could also record temperature of range of −45∼80°C and depth of up to 30 bar; however, only the measurements from the accelerometer, magnetometer and gyroscope were used for the analysis.

### Activity Measurements

One week prior to the feeding and escape response measurements, the length and mass of the fish were measured under anaesthesia induced with phenoxyethanol (<0.05%). A plastic plate (3×18 cm^2^) was sutured to the dorsal musculature just above the centre of mass (CM; 43% of the TL) using cable ties. The plate formed the base of the data logger. The position of the CM was determined by hanging a dead fish (different fish from those used for the activity measurements, FL = 61.8 cm) using a suture and needle [4]. The temporary attachment of the gyro logger to the base plastic plate was accomplished using cable ties; the fish were sedated using anaesthesia during this procedure. The mass of the data logger was less than 3% of the body mass of the fish. Although the size of the logger may be large for the fish, none of the animals showed any signs of stress after the tagging and quickly settled in the experimental tank.

In total, gyro loggers were attached to 5 fish. These fish were transferred from the holding tank to an identical and adjacent experimental tank (300 cm in diameter). The water level of the experimental tank was maintained at 0.44 m. The transfer and tagging time, including the time required for the anaesthesia, was less than 5 min. The fish were allowed to acclimate to the experimental tank for at least 12 hours prior to the start of the activity measurements. The data loggers were set to record the movements of the fish from 13∶00 to 15∶00 for 3∼5 consecutive days. During these 2 hours, between 3 and 10 live flathead silverside were introduced into the tank, and the fish were allowed to feed ad libitum. After the capture of several prey fish, at least a few hours or days were required for the fish to exhibit another feeding response to the presence of live prey. Consequently, the use of multiple scheduled recordings was considered appropriate to ensure the likelihood of recording a prey capture event under conditions of less stress to the fish. To obtain data on the escape movements of the fish, their escape responses were triggered by manually, randomly thrusting a PVC pole with a 100 cm×4.0 cm diameter to the bottom of the tank near the position of the fish when the fish were at least one body length from the tank wall. These responses were triggered at approximately 10 min intervals. Between 4 and 11 escape responses were recorded for each fish. RM of the fish were also recorded during the experiments.

The activities were also recorded with two video cameras that were located 2.85 m above the bottom of the tank: a 30 Hz standard USB webcam (HD C910, Logicool Co., Tokyo, Japan, with H264 Webcam 3.83 software, Timhillone software Co. Ltd., Nanshan, China), and a 200 Hz high-speed camera (HAS-L1, DITECT Co., Tokyo, Japan).

### Analysis

**Figure 1:**
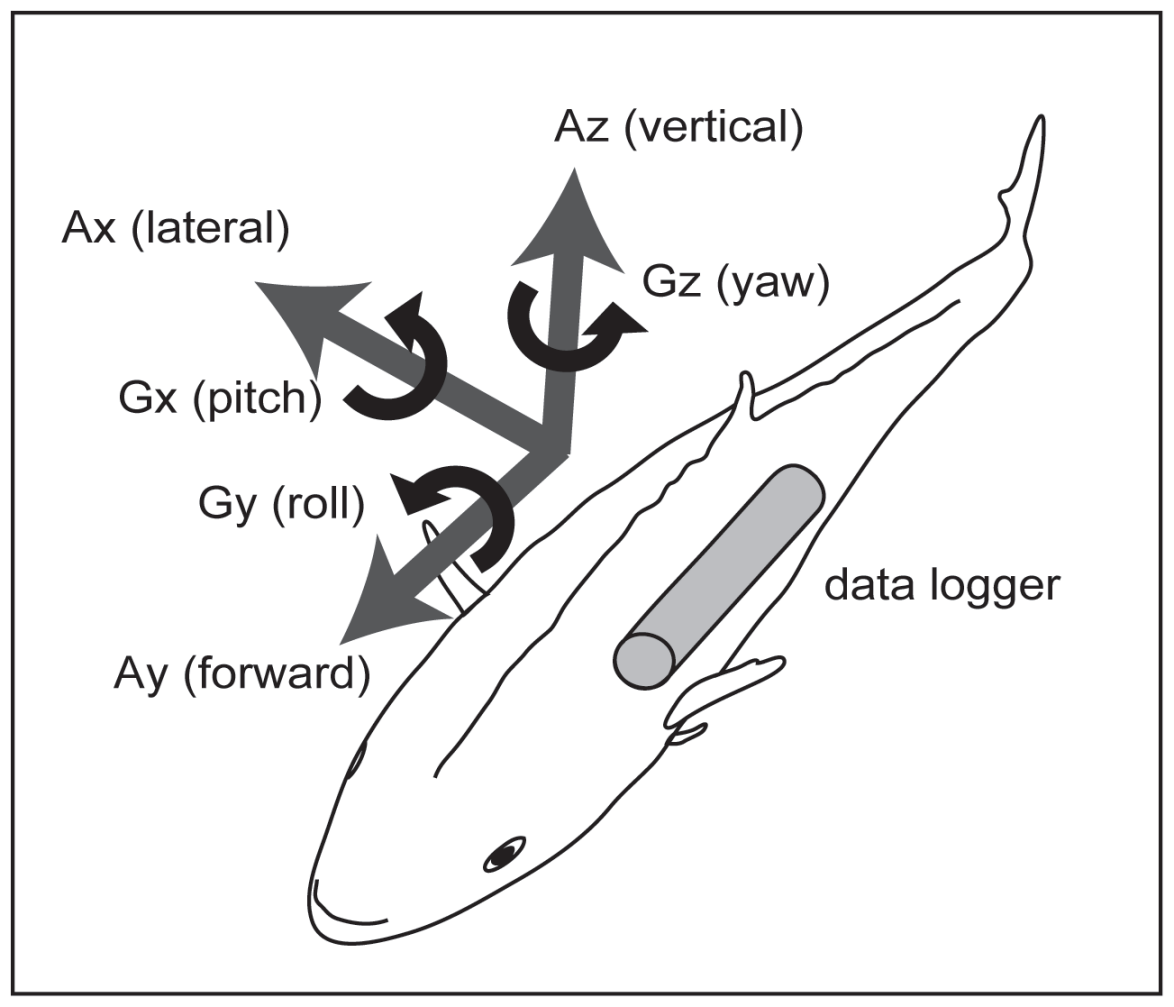
Schematic diagram of the fish coordinate frame. The directions of the acceleration (Ax, Ay, Az) and angular velocity (Gx, Gy, Gz) on the X-, Y- and Z-axes are shown.

To reconstruct locomotor variables from the sensor measurements, the orientation filter [24] was utilised, and dynamic acceleration and angular velocity were reconstructed [19]. The orientation filter merges the measurements of accelerometer, magnetometer and gyroscope to reconstruct attitude and dynamic acceleration using a gradient-decent algorithm (the original code is available at the author's website; http://www.x-io.co.uk/open-source-imu-and-ahrs-algorithms/). All of the locomotor variables were represented on the fish coordinate frame (Fig. 1). The programs that were used for the extraction of the data during FS and RM, and the reconstruction of the dynamic acceleration, attitude and locomotor variables from the data logger measurements were all created on Igor Pro 6.3 (WaveMetrics, Inc., Tigard, OR, USA). The statistical analysis was performed using R 2.15 (the R Foundation for Statistical Computing).

Characterization of FS behaviour recorded by the data logger was divided into two stages: 1. detection of the FS event and 2. classification of the type of FS movement (i.e., escape or feeding). The goal of this study was to establish the variables that can be used to accurately detect and classify the different types of FS behaviours.

The FS events that were monitored involved a large change in acceleration and angular velocity, which is very different from the changes that were observed in RM of the fish. Standard deviation (SD) of the magnitude of the acceleration (MA, vector summation of the acceleration in the x-, y- and z-directions) and the magnitude of the angular velocity (MG, vector summation of the angular velocity in the x-, y- and z-directions) were considered to detect the FS events. To investigate whether the differences between the FS and RM were statistically significant, some of the RM events of 1 s were extracted for each fish such that the total number of escape and feeding events and the number of RM were the same for each fish. Within each RM period, dash movements, observed in the video analysis, were selected as the RM dataset. All of the measurements of the escape and feeding responses were extracted for 1 s such that the maximum peak of the MA mapped to the centre of the duration. If both data of the RM and FS dataset were found to be normal by the Anderson-Darling test, Welch’s t test was used to determine the statistical significance of the difference between the RM and FS dataset; if the data were not normal, the Wilcoxon signed-rank test was used. Then, a sliding window analysis using the time-window of 1 s was applied to the entire recording dataset to calculate the SD of the MA and MG values for the period, and the optimal threshold value of each variable for detecting the FS events were established by a decision-tree algorithm (described later).

**Figure 2:**
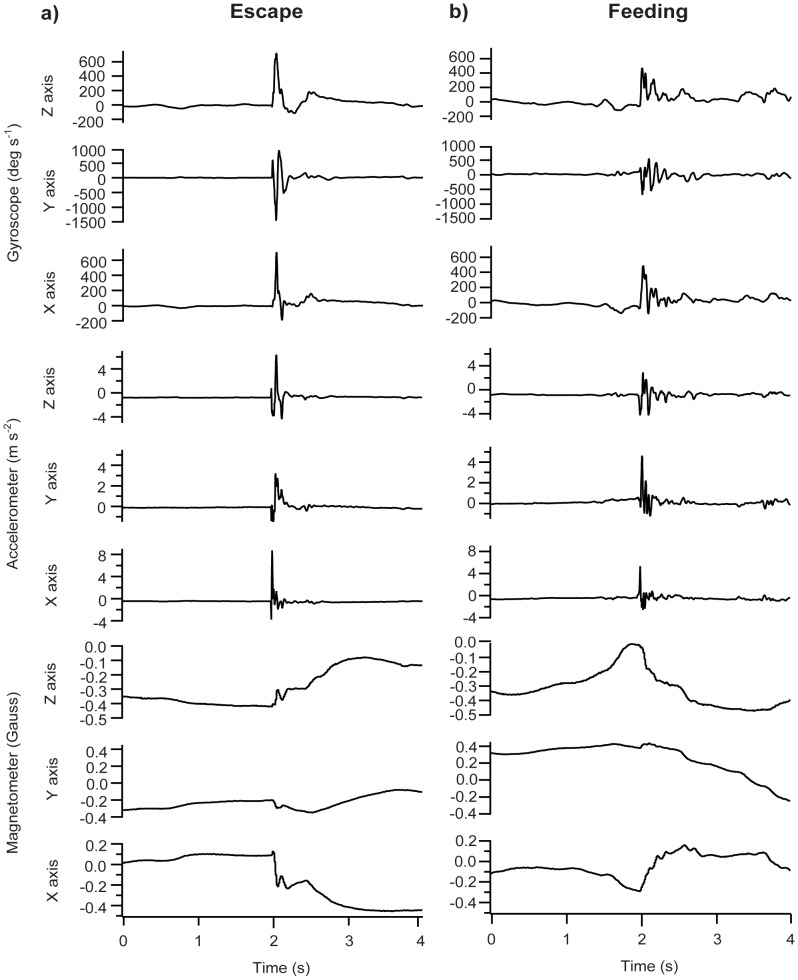
Example profiles of raw measurements by the data logger on a) escape and b) feeding. Measurements by the gyroscope, accelerometer and magnetometer on the X-, Y- and Z-axes are shown.

The classification of the escape and feeding movements was then studied (example profiles of the raw measurements by the gyro logger are shown in Fig. 2, and the data are available as datasets S1 and S2). First, the video images from a high-speed camera recording during the escape and feeding movements were observed to investigate the mechanical differences in the movements (sample movies are available as Movies S1 and S2). After detecting the FS events, the locomotor differences between the escape and feeding behaviour were then explored by using the values of the locomotor variables that were obtained through the gyro loggers. Two types of locomotor variables were calculated from the extracted 1 s FS dataset: axis-specific metrics and inter-axis metrics. Because the axis-specific variables were easier to interpret but may have been more affected by other factors, such as the attachment location of the data loggers on the fish, the motivation of the fish and environmental properties (e.g., temperature), the inter-axis metrics was also examined. The axis-specific metrics that were considered were the maximum, range, mean, SD, and root mean square (RMS) of the acceleration and angular velocity in the x-, y- and z-directions. The inter-axial metrics were the difference in the above axis-specific locomotor values along two different axes (e.g., x vs. y and x vs. z, which were calculated by the subtraction of the y and z values from the x values, respectively, and y vs. z, which was obtained through the subtraction of the z value from the y value). For a complete list of the variables and their abbreviations, please refer to the Table 2. The differences in the variable values between the escape and feeding movements were then identified, and the statistical significance of these differences was examined. As in the previous analysis, the normality of the data was determined using the Anderson-Darling test; the data that were found to follow a normal distribution were then analysed using Welch’s t test, and the data that were not normally distributed were studied using the Wilcoxon signed-rank test.

**Table 2:** Summary of the inter-axial variables (and their respective abbreviations) for classifying the types of FS behaviour (i.e., escape or feeding).

variable	between	abbreviation
difference of max acceleration (m s^−2^)	Ax v.s. Ay	DiffMaxAxAy
	Ay v.s. Az	DiffMaxAyAz
	Ax v.s. Az	DiffMaxAxAz
difference of range of acceleration (m s^−2^)	Ax v.s. Ay	DiffRangeAxAy
	Ay v.s. Az	DiffRangeAyAz
	Ax v.s. Az	DiffRangeAxAz
difference of mean acceleration (m s^−2^)	Ax v.s. Ay	DiffMeanAxAy
	Ay v.s. Az	DiffMeanAyAz
	Ax v.s. Az	DiffMeanAxAz
difference of SD of acceleration (m s^−2^)	Ax v.s. Ay	DiffSDAxAy
	Ay v.s. Az	DiffSDAyAz
	Ax v.s. Az	DiffSDAxAz
difference of RMS of acceleration (m s^−2^)	Ax v.s. Ay	DiffRMSAxAy
	Ay v.s. Az	DiffRMSAyAz
	Ax v.s. Az	DiffRMSAxAz
difference of max angular velocity(deg s^−1^)	Gx v.s. Gy	DiffMaxGxGy
	Gy v.s. Gz	DiffMaxGyGz
	Gx v.s. Gz	DiffMaxGxGz
difference of range of angular velocity(deg s^−1^)	Gx v.s. Gy	DiffRangeGxGy
	Gy v.s. Gz	DiffRangeGyGz
	Gx v.s. Gz	DiffRangeGxGz
difference of mean angular velocity(deg s^−1^)	Gx v.s. Gy	DiffMeanGxGz
	Gy v.s. Gz	DiffMeanGyGz
	Gx v.s. Gz	DiffMeanGxGz
difference of SD of angular velocity(deg s^−1^)	Gx v.s. Gy	DiffSDGxGy
	Gy v.s. Gz	DiffSDGyGz
	Gx v.s. Gz	DiffSDGxGz
difference of RMS of angular velocity(deg s^−1^)	Gx v.s. Gy	DiffRMSGxGy
	Gy v.s. Gz	DiffRMSGyGz
	Gx v.s. Gz	DiffRMSGxGz

Using the described locomotor variables and the resultant types of behaviour events, a decision tree (C4.5 algorithm) was constructed using a free machine learning software (WEKA 3; http://www.cs.waikato.ac.nz/ml/weka/) for the detection and classification of two types of FS movements. The threshold values of the decision tree for the detection and categorisation of the behaviour types were optimised (10). A ten-fold cross validation was performed, and the accuracy (precision, recall, and F-measure) was calculated as follows:
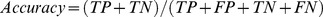
(1)

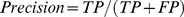
(2)

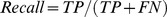
(3)

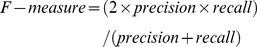
(4)where TP, FP, TN, and FN signify true positive, false positive, true negative, and false negative, respectively.

For the discrimination of the escape and feeding behaviour, to enhance the generalisability of the decision tree, it is important to select the minimum number of variables that can explain the differences between the movement types and can thus be used for the accurate classification of these different types. Therefore, the locomotor variables that had significantly different values (*α* = 0.05) between the escape and feeding behaviours, as was determined through a t test or Wilcoxon signed-rank test, were first selected. A CFS attribute subset evaluator [25], which is a correlation-based filter method, was used to select those variables that were highly correlated to the class attribute but exhibited a low correlation to each other. The final set of variables was selected by manually removing some of the variables until the highest accuracy was achieved.

The accuracy of the classification of the type of FS movement using different datasets was assessed. The datasets that were tested were the following: only accelerometer-derived variables (hereafter, acc-only); only gyroscope-derived variables (hereafter, gyro-only); and the combined variables, which were collected through the novel gyro method with an accelerometer, a magnetometer and a gyroscope (hereafter, all-combined). In the acc-only dataset, the gravity-based acceleration, which was simultaneously recorded with the dynamic acceleration in the accelerometer measurements, was not removed because its removal involves either the smoothing [26], [27] or low-pass filtering [28], [29] of the data, which induces variability in the values of the accelerometer-derived variables according to the level of smoothing or the filter limits selected [19].

## Results

A total of 41 escape (N = 11, 8, 11, 4 and 7 from 5 different fish) and 41 feeding events (N = 10, 11, 20, 0 and 0 from 5 different fish) was used in the analysis (Table 1). A total of 82 RM (21, 19, 31, 4 and 7 from 5 fish) was extracted for the statistical analysis between the RM and FS.

### Detection of FS Movements

Both of the SD values of the MA and MG were significantly higher (p<0.0001 for the MA and p<0.0001 for the MG) during the FS movements (11.77±0.53 m s^−2^ for the MA, and 229.22±9.30 deg s^−1^ for the MG) than during the RM (2.47±0.12 m s^−2^ for the MA, and 64.27±3.97 deg s^−1^ for the MG). Standard deviation of MA were able to detect the FS events with the precision rate of 0.98 and the recall rate of 0.96 using a threshold value of 5.16 m s^−2^. The SD of the MG were able to detect the FS events with the precision rate of 0.78 and the recall rate of 0.92 using a threshold value of 129.32 deg s^−1^. The precision and recall rate were higher using the SD of the MA than the SD of the MG.

### Classification of the Types of FS (Escape or Feeding) Movements

#### Video images

**Figure 3:**
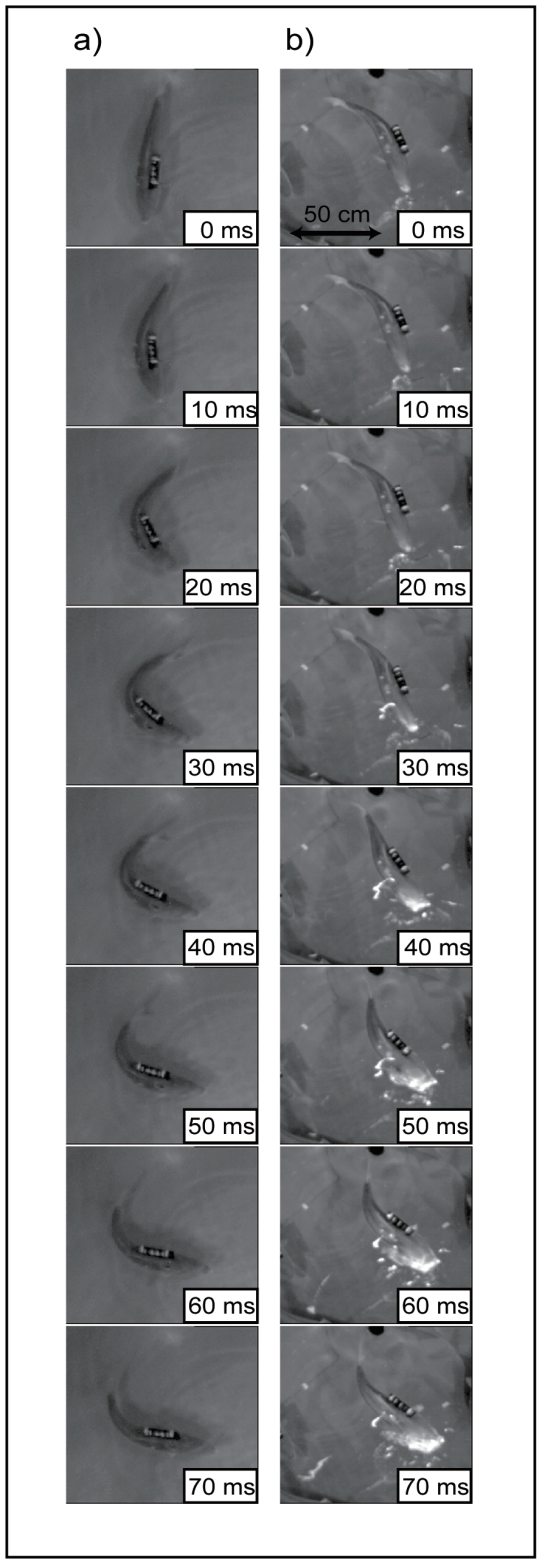
Example image sequences of typical a) escape and b) feeding movements. Full videos are available as the supporting information (Movies S1 and S2).

The video images showed that the fish typically bent their caudal fins at a larger angle and more rapidly during escape movements than during feeding (Fig. 3, for full videos, please see Movies S1 and S2). The feeding movements of the fish typically involved several tail undulations, which allowed the fish to place themselves in the direction of the evading target prey.

#### Axis-specific variables

**Figure 4:**
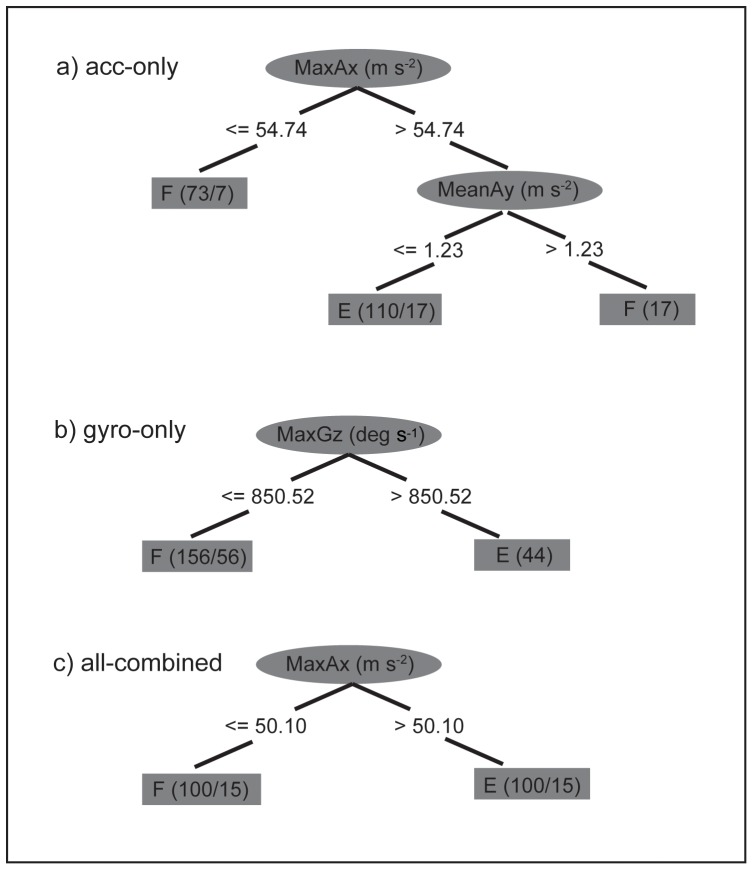
Decision trees for classifying escape or feeding movements by axis-specific variables. Three types of axis-specific variables were used to classify escape (E; N = 41) or feeding (F; N = 41) movements: a) acc-only, b) gyro-only, and c) all-combined datasets. The numbers in the box of the categories of FS indicate the percentage of events that were categorised into each type of movement and the percentage of these events that were miscategorised (after the diagonal).

**Figure 5:**
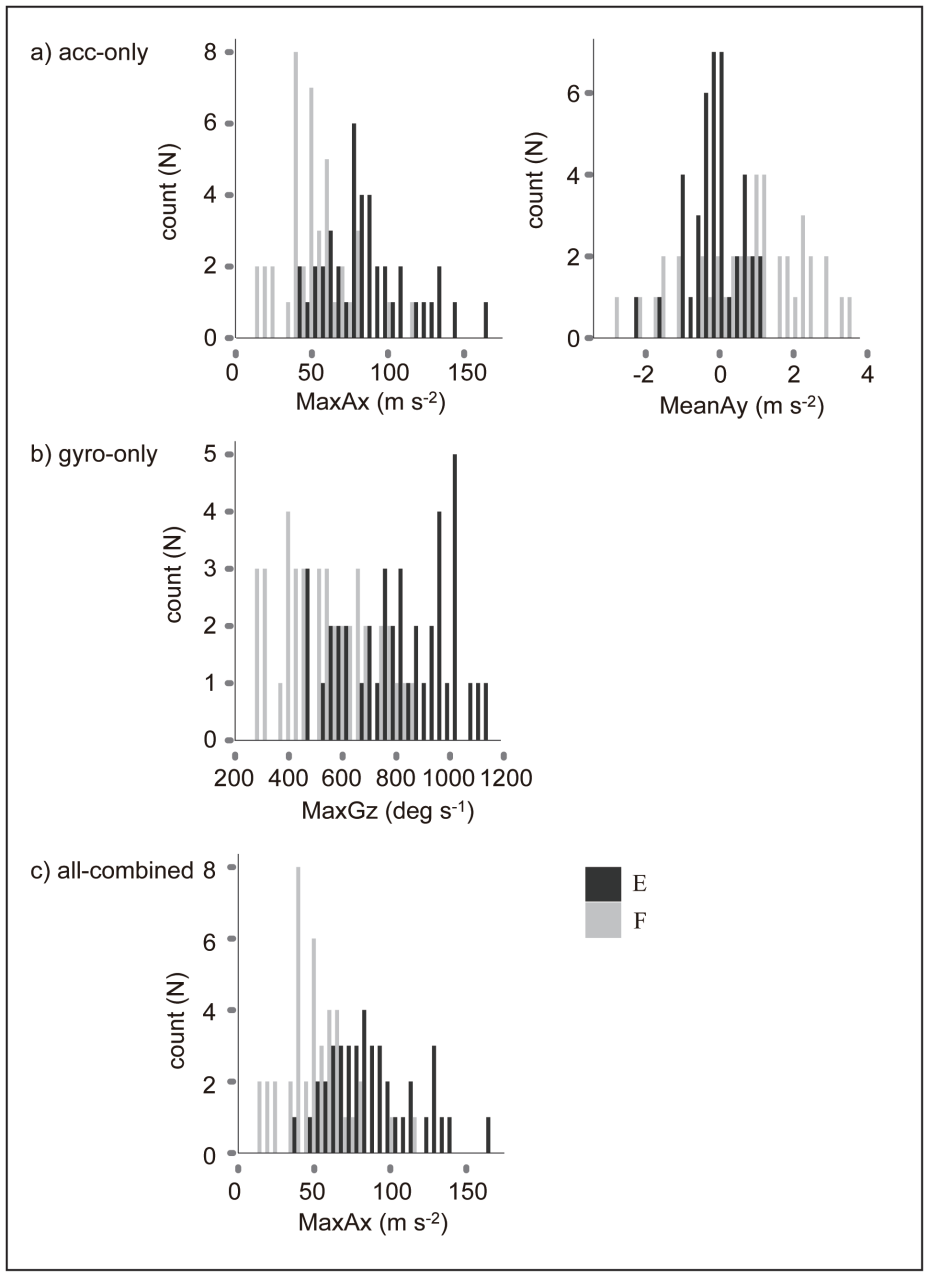
Histograms of axis-specific variables for classifying escape or feeding movements. Three types of axis-specific variables were used for the classification of the escape (N = 41) or feeding (N = 41) movements: a) acc-only, b) gyro-only, and c) all-combined datasets.

The previously described difference in the escape and feeding movements from the video observation was reflected in the axis-specific variables collected through the gyro loggers (Tables 3 and 4). The accuracy of the classification of the type of FS behaviour was highest when the maximum acceleration in the x-direction from the all-combined dataset (0.84) was used (Table 5, Fig. 4). The maximum acceleration in the x-direction and the mean acceleration in the y-direction from the acc-only dataset (0.78) and the maximum angular velocity in the z-direction (yaw direction) from the gyro-only dataset (0.62) exhibited the second- and third-highest accuracy, respectively (Table 5, Fig. 4). Note that the maximum acceleration in the acc-only dataset included the gravity-based acceleration, which was removed in the all-combined dataset with the aid of the gyroscope and magnetometer measurements. Therefore, the values of the maximum acceleration in the acc-only and all-combined datasets were different (Tables 3 and 4, Fig. 5).

**Table 3:** Summary of the means (sem) of the axis-specific variables derived from the all-combined or gyro-only datasets for classifying escape (N = 41) or feeding (N = 41) movements.

Variables	escape	feeding	Statistics^a^
MaxAx (m s^−2^)	89.05 (4.37)	49.04 (3.32)	p<0.0001****
MaxAy (m s^−2^)	46.99 (3.30)	32.10 (2.04)	p<0.001***
MaxAz (m s^−2^)	65.87 (3.33)	39.58 (2.34)	p<0.0001****
RangeAx (m s^−2^)	133.21 (6.01)	81.02 (5.05)	p<0.0001****
RangeAy (m s^−2^)	69.43 (4.30)	53.28 (3.51)	p<0.01**
RangeAz (m s^−2^)	112.56 (5.61)	70.27 (4.31)	p<0.0001****
MeanAx (m s^−2^)	0.05 (0.11)	0.00 (0.15)	NS^b^
MeanAy (m s^−2^)	0.61 (0.07)	0.58 (0.10)	NS
MeanAz (m s^−2^)	0.32 (0.05)	0.36 (0.08)	NS
SDAx (m s^−2^)	8.58 (0.46)	7.09 (0.51)	p<0.01**
SDAy (m s^−2^)	5.26 (0.31)	4.82 (0.30)	NS
SDAz (m s^−2^)	7.56 (0.36)	6.41 (0.44)	p<0.05*
RMSAx (m s^−2^)	8.60 (0.46)	7.16 (0.51)	p<0.01
RMSAy (m s^−2^)	5.31 (0.31)	4.87 (0.31)	NS
RMSAz (m s^−2^)	7.57 (0.36)	6.44 (0.44)	p<0.05*
MaxGx (deg s^−1^)	477.60 (32.84)	340.53 (17.98)	p<0.001***
MaxGy (deg s^−1^)	1298.94 (70.61)	962.76 (71.63)	p<0.01**
MaxGz (deg s^−1^)	814.68 (29.19)	530.88 (25.47)	p<0.0001****
RangeGx (deg s^−1^)	652.46 (38.29)	534.58 (26.98)	p<0.05*
RangeGy (deg s^−1^)	2367.95 (137.93)	1701.25 (132.55)	p<0.001***
RangeGz (deg s^−1^)	1145.68 (57.64)	841.51 (40.84)	p<0.0001****
MeanGx (deg s^−1^)	6.54 (2.50)	5.17 (3.66)	NS
MeanGy (deg s^−1^)	0.44 (1.22)	4.69 (2.39)	NS
MeanGz (deg s^−1^)	23.12 (6.92)	14.47 (10.59)	NS
SDGx (deg s^−1^)	63.75 (3.81)	65.29 (3.11)	NS
SDGy (deg s^−1^)	192.06 (12.20)	182.76 (16.18)	NS
SDGz (deg s^−1^)	138.78 (5.86)	139.67 (6.55)	NS
RMSGx (deg s^−1^)	65.80 (3.89)	68.89 (3.43)	NS
RMSGy (deg s^−1^)	192.16 (12.18)	183.44 (16.15)	NS
RMSGz (deg s^−1^)	146.91 (6.18)	154.53 (7.29)	NS

a t test or Wilcoxon singed-rank test was used.

b NS indicates no significance.

The acceleration variables were derived from the all-combined dataset, and the angular variables were derived from the all-combined or gyro-only datasets (which had the same values as the all-combined dataset). The statistical differences between the values of these variables during escape and feeding behaviours are also shown.

**Table 4:** Summary of the means (sem) of the axis-specific variables derived from the acc-only dataset for classifying escape (N = 41) or feeding (N = 41) movements.

variables	escape	feeding	Statistics^a^
MaxAx (m s^−2^)	87.82 (4.39)	50.40 (3.30)	p<0.0001****
MaxAy (m s^−2^)	46.65 (3.26)	33.33 (2.13)	p<0.01**
MaxAz (m s^−2^)	65.98 (3.11)	44.21 (2.24)	p<0.0001****
RangeAx (m s^−2^)	135.41 (6.11)	83.16 (5.09)	p<0.0001****
RangeAy (m s^−2^)	69.98 (4.26)	53.38 (3.54)	p<0.01**
RangeAz (m s^−2^)	114.17 (5.66)	70.43 (4.32)	p<0.0001****
MeanAx (m s^−2^)	−2.16 (0.31)	−0.92 (0.38)	p<0.05*
MeanAy (m s^−2^)	−0.08 (0.11)	0.75 (0.24)	p<0.01**
MeanAz (m s^−2^)	−8.72 (0.07)	−8.45 (0.11)	NS^b^
SDAx (m s^−2^)	8.89 (0.46)	7.48 (0.53)	p<0.01**
SDAy (m s^−2^)	5.41 (0.30)	5.16 (0.31)	NS
SDAz (m s^−2^)	7.71 (0.36)	6.46 (0.44)	p<0.01**
RMSAx (m s^−2^)	9.41 (0.43)	8.02 (0.49)	p<0.01**
RMSAy (m s^−2^)	5.45 (0.30)	5.43 (0.31)	NS
RMSAz (m s^−2^)	11.77 (0.25)	10.88 (0.28)	p<0.01**

a t test or Wilcoxon singed-rank test was used.

b NS indicates no significance.

The statistical differences between the values of these variables during escape and feeding behaviours are also shown.

**Table 5:** Summary of the classification rate of escape and feeding movements using the axis-specific variables.

variabletype	category	accuracy	precision	recall	F-measure	variables	
acc-only	escape	0.78	0.76	0.83	0.79	MaxAx	MeanAy
	feeding		0.81	0.73	0.77		
gyro-only	escape	0.62	0.63	0.61	0.62	MaxGz	
	feeding		0.62	0.63	0.63		
all-combined	escape	0.84	0.83	0.85	0.84	MaxAx	
	feeding		0.85	0.83	0.84		

The variables used for the classification are also shown.

The decision trees and histograms showed that 66% and 85% of the feeding movements had a smaller maximum acceleration in the x-direction in the acc-only and all-combined datasets respectively, whereas the escape movements had larger values (Figs. 4 and 5). The mean acceleration in the y-direction of the acc-only dataset could be used to divide the FS movements that had a larger maximum acceleration in the x-direction into escape and feeding movements (the feeding movements had a larger mean acceleration in the y-direction) (Figs. 4 and 5). In addition, the maximum angular velocity in the z-direction of the gyro-only dataset was larger during 44% of the escape movements (Figs. 4 and 5).

#### Inter-axial variables

**Figure 6:**
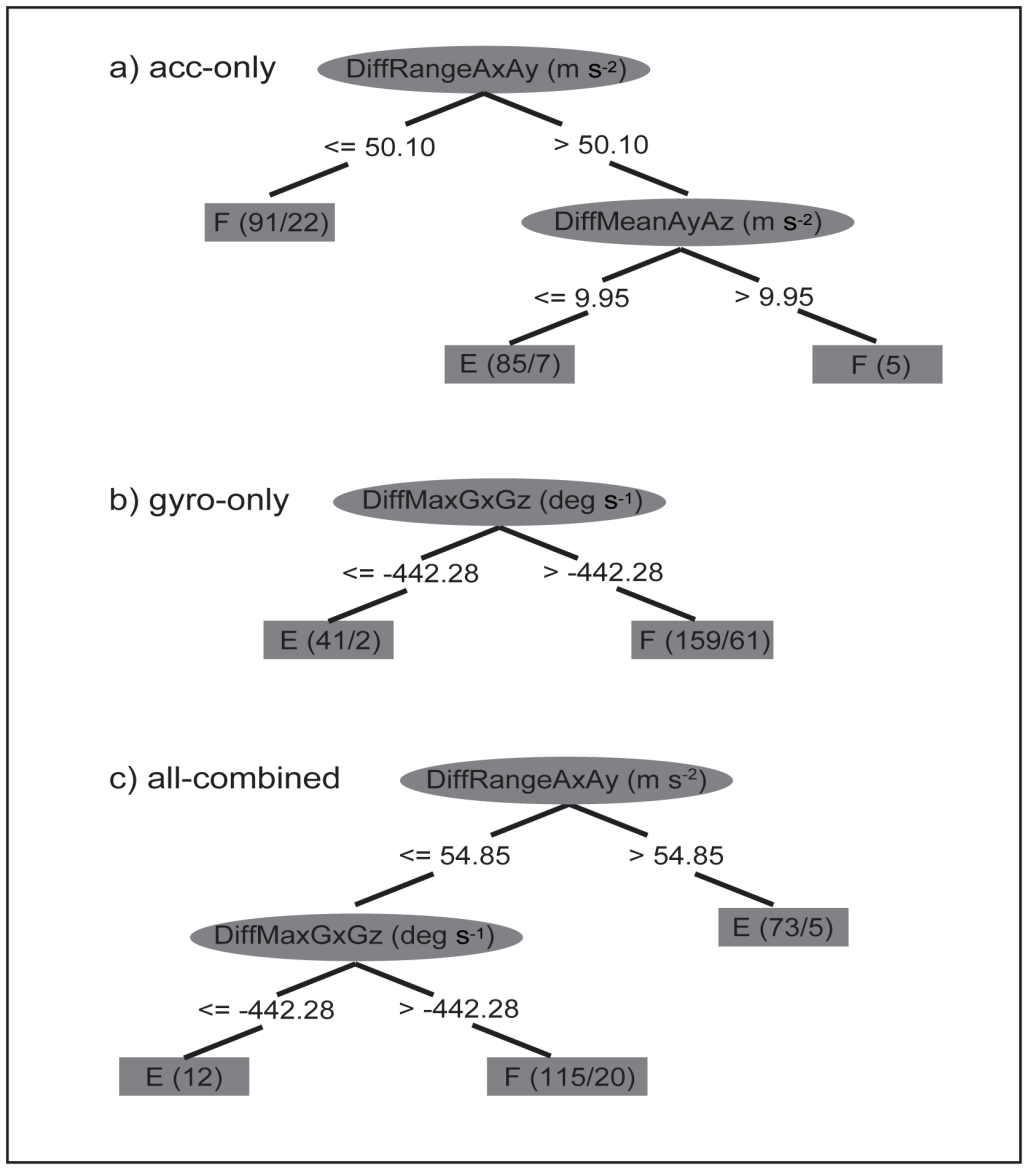
Decision trees for classifying escape or feeding movements by inter-axial variables. Three types of inter-axial variables were used for the classification of the escape (N = 41) or feeding (N = 41) movements: a) acc-only, b) gyro-only, and c) all-combined datasets. The numbers in the box of the categories of FS indicate the percentage of events that were categorised into each movement type and the percentage of these events that were miscategorised (after the diagonal).

The difference in the escape and feeding movements was also reflected in the inter-axial variables (Tables 6 and 7). The accuracy of the classification of the types of FS movements was highest (0.84) when the difference of the range of the acceleration between the x- and y-directions and the difference of the maximum angular velocity in the x- and z-directions from the all-combined dataset were used (Table 8, Fig. 6). The differences in the range of the acceleration in the x- and y-directions and in the mean acceleration in the y- and z-directions from the acc-only dataset (0.80) and the difference in the maximum angular velocity in the x- and z-directions from the gyro-only dataset (0.66) exhibited the second- and third-highest accuracy, respectively (Table 8, Fig. 6). Since the all-combined dataset produced the highest accuracy, overall accuracy, combining the detection rate of the FS behaviour using the SD of the MA and the identification rate of the type of FS behaviour using the all-combined variables, was evaluated as (the number of accurately classified instances of feeding and escape)/(the number of instances detected as the FS behaviour) and (the number of accurately classified instances of feeding and escape)/(the number of instances of the FS behaviour), which were 0.83 and 0.83 respectively.

**Table 6:** Summary of the means (sem) of the inter-axial variables dereived from the all-combined or gyro-only datasets for classifying escape (N = 41) or feeding (N = 41) movements.

Variables	escape	feeding	Statistics^a^
DiffMaxAxAy (m s^−2^)	42.06 (4.19)	16.94 (2.71)	p<0.0001****
DiffMaxAyAz (m s^−2^)	−18.88 (2.86)	−7.48 (2.32)	p<0.01**
DiffMaxAxAz (m s^−2^)	23.18 (3.80)	9.46 (2.52)	p<0.01**
DiffRangeAxAy (m s^−2^)	63.77 (4.64)	27.74 (3.74)	p<0.0001****
DiffRangeAyAz (m s^−2^)	−43.13 (4.55)	−16.98 (3.94)	p<0.0001****
DiffRangeAxAz (m s^−2^)	20.64 (4.50)	10.75 (3.72)	NS^b^
DiffMeanAxAy (m s^−2^)	−0.57 (0.13)	−0.58 (0.19)	NS
DiffMeanAyAz (m s^−2^)	0.29 (0.08)	0.22 (0.11)	NS
DiffMeanAxAz (m s^−2^)	−0.28 (0.11)	−0.36 (0.18)	NS
DiffSDAxAy (m s^−2^)	3.32 (0.23)	2.27 (0.27)	p<0.01**
DiffSDAyAz (m s^−2^)	−2.30 (0.23)	−1.59 (0.26)	p<0.05*
DiffSDAxAz (m s^−2^)	1.02 (0.27)	0.68 (0.29)	NS
DiffRMSAxAy (m s^−2^)	3.29 (0.23)	2.29 (0.26)	p<0.01**
DiffRMSAyAz (m s^−2^)	−2.26 (0.23)	−1.57 (0.26)	p<0.05*
DiffRMSAxAz (m s^−2^)	1.03 (0.27)	0.72 (0.29)	NS
DiffMaxGxGy (deg s^−1^)	−821.34 (60.99)	−622.23 (66.56)	p<0.05*
DiffMaxGyGz (deg s^−1^)	484.26 (66.7)	431.88 (67.71)	NS
DiffMaxGxGz (deg s^−1^)	−337.08 (34.88)	−190.35 (23.54)	p<0.001***
DiffRangeGxGy (deg s^−1^)	−1715.49 (120.19)	−1166.67 (120.46)	p<0.01**
DiffRangeGyGz (deg s^−1^)	1222.27 (116.58)	859.74 (125.18)	p<0.05*
DiffRangeGxGz (deg s^−1^)	−493.22 (44.59)	−306.93 (34.39)	p<0.01**
DiffMeanGxGy (deg s^−1^)	6.10 (2.44)	0.48 (5.15)	NS
DiffMeanGyGz (deg s^−1^)	−22.67 (6.66)	−9.78 (11.87)	NS
DiffMeanGxGz (deg s^−1^)	−16.57 (5.38)	−9.30 (8.71)	NS
DiffSDGxGy (deg s^−1^)	−128.31 (11.74)	−117.47 (15.65)	NS
DiffSDGyGz (deg s^−1^)	53.28 (10.25)	43.06 (14.88)	NS
DiffSDGxGz (deg s^−1^)	−75.03 (5.17)	−74.41 (5.15)	NS
DiffRMSGxGy (deg s^−1^)	−126.36 (11.93)	−114.54 (15.78)	NS
DiffRMSGyGz (deg s^−1^)	45.26 (10.39)	28.91 (15.28)	NS
DiffRMSGxGz (deg s^−1^)	−81.11 (5.43)	−85.64 (5.75)	NS

a t test or Wilcoxon singed-rank test was used.

b NS indicates no significance.

The acceleration variables were derived from the all-combined dataset and the angular variables were derived from the all-combined or gyro-only datasets (which had the same values as the all-combined dataset). The statistical differences between the values of these variables during escape and feeding behaviours are also shown.

**Table 7:** Summary of the means (sem) of the inter-axial variables derived from the acc-only dataset for classifying escape (N = 41) or feeding (N = 41) movements.

variables	escape	feeding	Statistics^a^
DiffMaxAxAy (m s^−2^)	41.17 (4.13)	17.07 (2.67)	p<0.0001****
DiffMaxAyAz (m s^−2^)	−19.33 (2.78)	−10.88 (2.42)	p<0.01**
DiffMaxAxAz (m s^−2^)	21.84 (3.90)	6.19 (2.54)	p<0.01**
DiffRangeAxAy (m s^−2^)	65.43 (4.70)	29.78 (3.8)	p<0.0001****
DiffRangeAyAz (m s^−2^)	−44.19 (4.69)	−17.05 (3.96)	p<0.0001****
DiffRangeAxAz (m s^−2^)	21.24 (4.58)	12.73 (3.71)	NS^b^
DiffMeanAxAy (m s^−2^)	−2.08 (0.31)	−1.67 (0.43)	NS
DiffMeanAyAz (m s^−2^)	8.64 (0.12)	9.20 (0.22)	p<0.05*
DiffMeanAxAz (m s^−2^)	6.56 (0.33)	7.52 (0.43)	NS
DiffSDAxAy (m s^−2^)	3.48 (0.24)	2.32 (0.28)	p<0.01**
DiffSDAyAz (m s^−2^)	−2.30 (0.23)	−1.29 (0.25)	p<0.01**
DiffSDAxAz (m s^−2^)	1.18 (0.28)	1.03 (0.31)	NS
DiffRMSAxAy (m s^−2^)	3.95 (0.22)	2.60 (0.26)	p<0.0001****
DiffRMSAyAz (m s^−2^)	−6.32 (0.21)	−5.45 (0.21)	p<0.01**
DiffRMSAxAz (m s^−2^)	−2.37 (0.31)	−2.85 (0.34)	NS

a t test or Wilcoxon singed-rank test was used.

b NS indicates no significance.

The statistical differences between the values of these variables during escape and feeding behaviours are also shown.

**Table 8:** Summary of the classification rate of escape and feeding movements using the inter-axial variables.

variable type	category	accuracy	precision	recall	F-measure	variables	
acc-only	escape	0.80	0.82	0.78	0.80	DiffRangeAxAy	DiffMeanAyAz
	feeding		0.79	0.83	0.81		
gyro-only	escape	0.66	0.88	0.37	0.52	DiffMaxGxGz	
	feeding		0.60	0.95	0.74		
all-combined	escape	0.84	0.89	0.78	0.83	DiffRangeAxAy	DiffMaxGxGz
	feeding		0.80	0.90	0.85		

The variables used for the classification are also shown.

**Figure 7:**
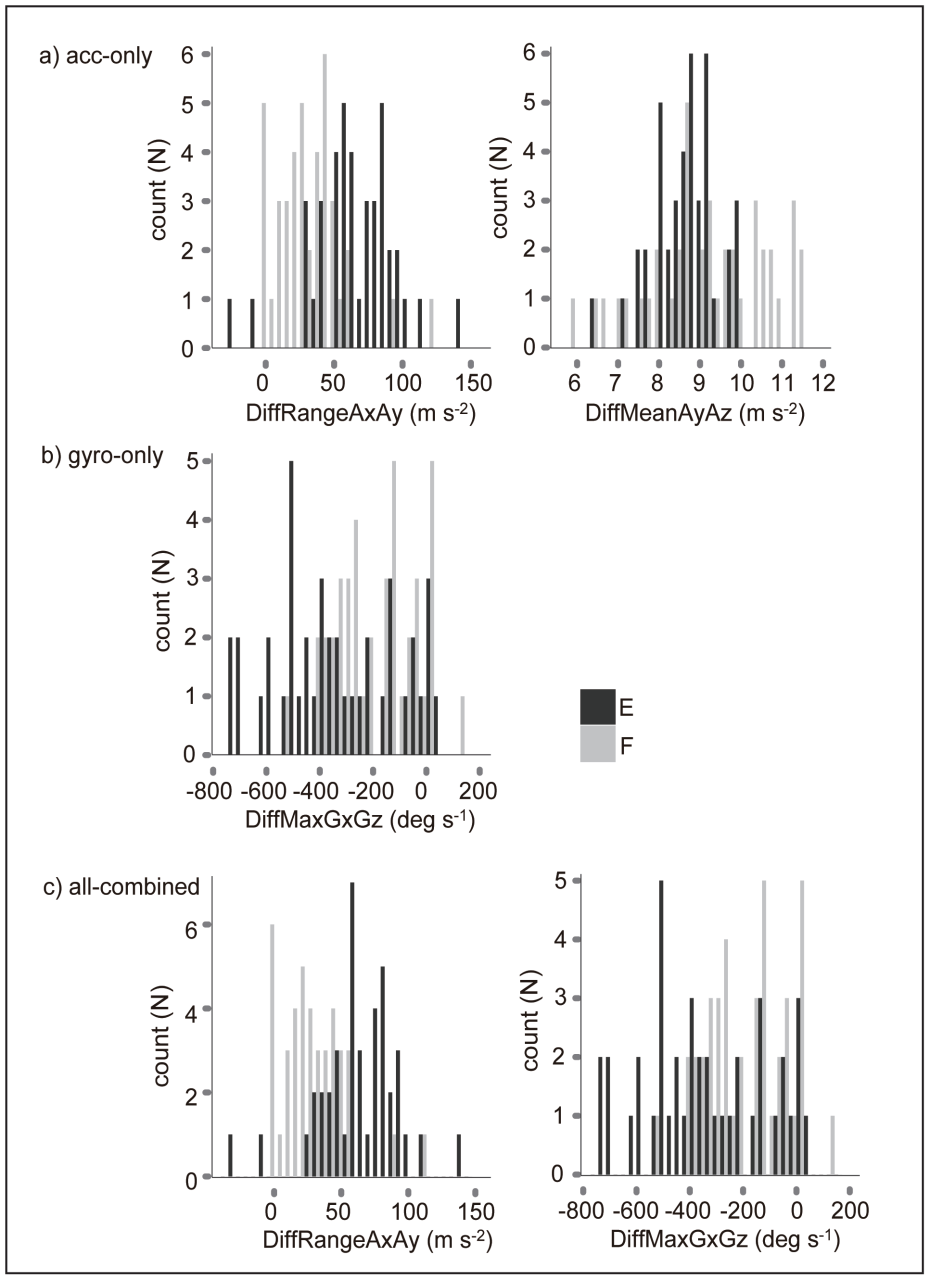
Histograms of the inter-axial variables for classifying escape or feeding movements. Three types of inter-axial variables were used for the classification of the escape (N = 41) or feeding (N = 41) movements: a) acc-only, b) gyro-only, and c) all-combined datasets.

The decision trees and histograms showed that 78% and 68% of the escape movements in the acc-only and all-combined dataset respectively had a larger difference in the range of the acceleration in the x- and y-directions, which meant that the range of acceleration in the x-direction was usually larger than the acceleration in the y-direction during escape movements (Figs. 6 and 7); consequently, this difference was smaller during feeding movements (Figs. 6 and 7). In the acc-only dataset, the difference of the mean acceleration in the y- and z-directions divided the movements into 78% of the escape movements and in only 5% of the feeding movements, which means that mean acceleration in the y-direction tended to be larger than in the z-direction during the feeding movements (Figs. 6 and 7). In the gyro-only and all-combined datasets, the difference of the maximum angular velocity in the x- and z- directions was larger during feeding than during escape behaviours, which meant that during escape movements, the maximum angular velocity in the z-direction tended to be larger than the angular velocity in the x-direction (note that the difference of the maximum angular velocity between the x- and z-directions was calculated through the subtraction of the z-direction value from the x-direction value) (Figs. 6 and 7).

## Discussion

In this study, the monitoring of FS movements of a cruiser fish by the gyro logger was attained through two processes: 1. the detection of FS events and 2. the classification of the type of FS behaviour (i.e., escape or feeding). The classification of whether a FS event is part of an escape or feeding behaviour is important because these two movements typically involve a high acceleration and a high degree of rotation and thus have similar acceleration and angular velocity profiles. Therefore, to accurately classify the type of FS movement, the detailed locomotor variables that were obtained through the gyro logger measurements during escape and feeding movements were explored.

From the decision trees, the detection of FS movements was possible with high precision and recall rate through the use of the SD of the MA and with relatively high precision and recall rate through the use of the SD of the MG because FS movements have higher acceleration and angular velocity variations than RM. This result showed that accelerometer alone can be used to more accurately detect these FS movements than gyroscope alone. Because the SD, which indicates the variation, was utilised instead of the maximum, mean and RMS (the range value is the same as the maximum value in this case because the minimum MA and MG value is zero), the thresholds for the decision-tree categorisation are probably less prone to be affected by other factors, such as the attachment location of the data logger, the motivation of the fish, and environmental properties (e.g., temperature).

To classify escape or feeding movements, the axis-specific variables were first investigated. The differences in the acceleration and angular velocity variables between escape and feeding movements indicate the different strategies that are used in the different behaviours. The decision trees and histograms showed that feeding movements typically had a smaller maximum acceleration in the x-direction than that observed in the escape movements, which reflects the lower intensity of the beating of the caudal fin during feeding than escape behaviour, as shown in the video images. This difference may occur because feeding requires a larger degree of trajectory adjusting (e.g., manoeuvrability and accuracy) to follow the evading prey [30]; furthermore, fish do not use a large amount of power, which may decrease the amount of their control in the movement direction. Escape movements, however, may require that the fish place a higher priority on its quick separation from threats, which requires less control in the movement direction. Although the movement and species of the prey, the direction of refuge and stimulus, and the position of other individuals in the school may change the direction of the movement (reviewed by [31], [32]), the manner of caudal fin movement may be less variant depending on the context. In the acc-only dataset, the higher mean acceleration in the y-direction categorised the movements with a larger maximum acceleration in the x-direction into feeding behaviour, which indicates that feeding movements occasionally require a longer chasing period before the final attack. In these cases, the intensity of the forward acceleration was maintained at a high level. Since escape does not typically involve a movement that requires a constant high acceleration, as does chasing, these movements typically have a smaller mean acceleration in the y-direction. In the gyro-only dataset, the maximum angular velocity in the yaw direction was typically larger during escape than during feeding, although there were overlaps in the distributions. This difference may also be due to the different strategies that are involved in the movements, where feeding requires the adjustment of movement to capture the evading prey, which requires a smaller change rate in direction, whereas escape requires a considerable amount of turning, to avoid threats.

Even though the all-combined dataset had the same variable (maximum acceleration in the x-direction) as the acc-only dataset, the accuracy of the classification of escape and feeding behaviour was higher when the all-combined dataset was used. In the all-combined dataset, the gravity-based acceleration was removed, and the maximum acceleration reflected the accurate dynamic acceleration [19]. The gravity-based acceleration was not removed in the acc-only dataset because the smoothing [26], [27] or low-pass filtering [28], [29] methods that would produce variability in the acceleration variables. The ratio of the gravity-based acceleration to the dynamic acceleration in the accelerometer measurements can be high when the acceleration intensity is low. This gravity-based acceleration reflects the change of attitude in roll and pitch movements. Therefore, the maximum acceleration has different meanings in the all-combined and acc-only datasets. An accurate value of the maximum acceleration may be important for the classification of these two types of movement. Furthermore, the accuracy of the classification was highest when the all-combined dataset was used than if the acc-only or gyro-only datasets were utilised, which suggests that the data that can be collected using an accelerometer or a gyroscope alone lack some information that is required for the classification of the different types of FS movements. Consequently, the combination of an accelerometer, a gyroscope and a magnetometer is important.

Although the axis-specific variables were easier to interpret, the threshold values may be affected by other factors, such as the attachment location of the data loggers, the motivation of the fish, and environmental properties (e.g., temperature). Therefore, the inter-axial variables were also considered because these reflect the relative differences of movement in the different directions (x, y and z). As with the axis-specific variables, the accuracy of the classification of escape or feeding movements by inter-axial variables was highest when the all-combined dataset was used. The variables in the all-combined dataset were also utilised by the acc-only and gyro-only datasets. The difference in the range of the acceleration in the x- and y-directions was typically larger during escape movements. This may be because feeding requires the adjustment of trajectories to capture the evading prey, which involves an increased amount of wiggle movements and a smaller change in the lateral direction by the beating of the caudal fin. This would result in the smaller difference in the range of acceleration in the x- and y-directions, as described with the axis-specific variables. Escape movements, however, may require a larger degree of caudal fin beating to quickly increase the distance from threats; consequently, these movements exhibit a larger lateral acceleration in both the positive and negative directions, which results in a greater degree of acceleration in the x-direction than in the y-direction. The difference in the mean acceleration in the y- and z-directions showed that a few feeding events have a large difference in the acceleration in the y- and z-directions, which may reflect the duration of the chase during which the forward acceleration is maintained at a higher level than the vertical acceleration. The difference in the maximum angular velocity in the x- and z-directions showed that the maximum angular velocity in the z-direction (yaw) was larger than in the x-direction (pitch) during escape movements, whereas this difference was closer to zero in feeding movements. This result may indicate that escape movements involve responses that have a higher maximum angular velocity in the z-direction (i.e., turning) and that the change in the pitch direction is not important during this type of movement. Because feeding may require the adjustment of the movement direction to follow the movement of the prey, the rate of change in the yaw direction is expected to be lower than during escape movements, whereas the change in the pitch direction is more important in these cases.

We have established locomotor variables and the threshold values to detect and identify the escape and feeding behaviours of a cruise fish in laboratory experiments. It is possible that the movement signatures of fish in a controlled condition may be different from those in the field [33]. In this regard, it would be ideal to have the locomotor variables and especially the threshold values calibrated in the field using animal-borne motion sensors and video cameras simultaneously, if these experiments are feasible. Nevertheless, our study in laboratory settings will be useful for future research in the open water. In the open water, where the fish is not restricted in its depth movements, it may swim at steep angles and significantly change swimming angles. However, it should move along the principal axes of its coordinate frame while the fish coordinate frame simultaneously changes in the earth coordinate frame when the fish changes its posture. Therefore, if the change rate of angle and translational movement (i.e. angular velocity and dynamic acceleration) is utilized and represented in the fish coordinate frame (not the earth coordinate frame), it is unlikely that the movement signatures are affected by the steep angle of posture during their depth change. For the experiment in the open water, a data logger can be recovered from the tagged fish using the time-scheduled release system [34].

The method developed in this study can be used to monitor feeding and escape behaviours, or even other complicated bahaviours, of other animals with a high accuracy, by recording the detailed movements with a high sampling frequency through a gyro logger. While the relationship between the sampling frequency and the accuracy of identifying the fast-start behavior is not the focus of this study, 500 Hz sampling frequency as used in our experiments may be unnecessary. However, sufficiently high sampling frequency (e.g. 100 or 200 Hz) is needed to avoid a serious temporal aliasing in the data of fast-track movements that usually last only a fraction of a second [16]. Since the sampling frequency is related to the longevity of recording hours with limited battery and memory (and even the size of the data logger), the sampling frequency should be adjusted to objective behaviours. In the future, it will be important to develop a smaller gyro logger that can be implanted in the body cavity of animals to remove the hydrodynamic drag of the devices. At the same time, implementation of on-board processing, to boot the device and start recording only when important events are happening, will be needed to create a device that can monitor long-term behaviours [35]. To achieve that, important movement variables and the thresholds for monitoring specific behaviours must be firstly identified as done in our study.

## Supporting Information

Dataset S1 **Raw measurements of escape movement by the gyro logger.**
(TXT)

Dataset S2 **Raw measurements of feeding movement by the gyro logger.**
(TXT)

Movie S1 **Escape movie.** Each frame obtained through the 200 Hz high speed camera was recombined to produce a 10 Hz movie (One second in the video time corresponds to 0.05 s in a real time).
(MOV)

Movie S2 **Feeding movie.** Each frame obtained through the 200 Hz high speed camera was recombined to produce a 10 Hz movie (One second in the video time corresponds to 0.05 s in a real time).
(MOV)
